# Short (seven days) versus standard (fourteen days) oestrogen administration in a programmed frozen embryo transfer cycle: a retrospective cohort study

**DOI:** 10.1186/s13048-022-00967-5

**Published:** 2022-03-21

**Authors:** Wen-Jing Jiang, Jing-Yan Song, Zhen-Gao Sun

**Affiliations:** 1 The First Clinical College, Shandong University of Traditional Chinese Medicine, Jinan, China; 2 Reproductive and Genetic Centre of Integrated Medicine, The Affiliated Hospital of Shandong University of Traditional Chinese Medicine, Jinan, China

**Keywords:** Endometrial preparation, Oestrogen, Frozen-thawed embryo transfer, Cumulative live birth rate, Maternal and neonatal complications

## Abstract

**Research question:**

What influence does seven days of oestrogen administration versus fourteen days have on the reproductive outcomes of frozen-thawed embryo transfer (FET) in programmed endometrial preparation cycles?

**Design:**

In a retrospective study, conducted at a university-affiliated tertiary hospital, a total of 2628 infertile patients (4142 FET cycles) were divided into one of two groups between January 2014 and December 2020: group A (*n* = 1406, seven days of oestrogen before progesterone (P4) supplementation) and group B (*n* = 2716, fourteen days of oestrogen before P4 supplementation). The primary outcome was cumulative live birth rate (CLBR). Secondary outcomes were other pregnancy-related outcomes, maternal and neonatal complications.

**Results:**

No significant difference in CLBR was observed when comparing seven versus fourteen days of oestrogen administration before starting P4 supplementation (47.6% vs. 48.8%, *P* = 0.537). Furthermore, multivariable logistic regression analysis revealed that oestrogen administration in programmed FET cycles (7 days vs. 14 days) was not significantly associated with CLBR (OR 1.04, 95% CI 0.89–1.23). The risks of maternal and neonatal complications were comparable between the two groups.

**Conclusions:**

Variation in the duration of oestradiol supplementation before P4 initiation does not impact FET reproductive outcomes. For infertile women who desire to conceive as soon as feasible, short (seven days) oestrogen administration in a programmed FET cycle may be a suitable alternative.

**Supplementary Information:**

The online version contains supplementary material available at 10.1186/s13048-022-00967-5.

## Introduction

In recent years, with the rapid development of assisted reproductive technology (ART) and the continuous progress of vitrification technology, FET technology has become the mainstream. FET plays an important role in preventing ovarian hyperstimulation syndrome, preserving the remaining embryos, increasing the cumulative pregnancy rate, and reducing ectopic pregnancy [1]. Studies have found that the clinical pregnancy rate of FET is similar to or even higher than that of fresh embryo transfer [2].

Before undergoing FET, the endometrium is mostly prepared clinically using natural cycles, programmed cycles, and ovulation induction cycle protocols [3]. Endometrial preparation and its synchrony with embryonic developmental stages are essential factors to ensure the maximization of endometrial receptivity and the quality of embryo implantation [4]. Programmed cycles offer greater flexibility for scheduling transfer without reducing live birth rates compared to natural and ovulation induction cycles, and are therefore more widely used clinically [5].

Previous studies have confirmed that different routes of oestrogen administration in programmed cycles do not affect the clinical outcome of patients [7–9]. However, these studies did not address whether the timing of exposure to oestrogen during the endometrial preparation phase would have an impact on clinical outcomes. In studies of non-genetically tested embryo transfer, it has been shown that prolonged exposure to oestrogen, i.e., > 32 days, will result in significantly lower live birth rates and increased miscarriage rates after autologous frozen-thawed blastocyst transfer [10]. However, in a study of euploid embryos, Sekhon et al. did not find a link between exposure time to oestrogen and clinical outcomes (pregnancy and miscarriage rates) [11]. In addition, the optimal duration of oestrogen when preparing the endometrium in a programmed cycle has not yet been defined.

Therefore, this study retrospectively assessed the relationship between oestrogen duration and reproductive outcomes in programmed cycle frozen embryo transfer, in order to provide more options for patients who wish to become pregnant as soon as possible.

### Materials and methods

#### Study design

Between January 2014 and December 2020, individuals treated at the authors' reproductive clinic completed autologous in vitro fertilization (IVF) / intracytoplasmic sperm injection (ICSI) cycles and had supernumerary embryos cryopreserved for future FET were included in this single-centre retrospective cohort study. The study was authorized by the local institutional review board (Reproductive Ethics Committee of The Affiliated Hospital of Shandong University of Traditional Chinese Medicine, approval no. SDTCM/E2110-03, dated 11 October 2021) and was undertaken at a public tertiary referral university hospital.

### Eligibility criteria

This study only included patients who had autologous programmed cycle FETs after performing a freeze-all strategy or after failed fresh embryo transfer attempts. This study was excluded for the following patients: (1) patients aged ≥ 45 years at the time of oocyte retrieval, (2) patients previously diagnosed with congenital or acquired uterine abnormalities, (3) patients undergoing blastocyst biopsy for preimplantation genetic testing (PGT) or preimplantation genetic diagnosis (PGD), (4) patients who underwent in vitro maturation (IVM), (5) patients who had ≥ 3 embryos transferred, (6) patients who used embryos derived from previous stimulation cycles (i.e., cryopreserved oocytes and/or donor oocytes), (7) patients who were unable to achieve an endometrial thickness of 8 mm within 7 or 14 days of initiating oestrogen supplementation, and (8) patients who required oestrogen administration beyond the standard oral regimen used. Additionally, natural cycle FETs that did not involve the administration of oestrogen or P4 were excluded. The included patients were divided into one of two groups: Group A (7 days of oestrogen prior to P4 supplementation) and Group B (14 days of oestrogen prior to P4 supplementation). Patient assignment to treatment was based simply on endometrial thickness meeting criteria above 7 mm and no follicular luteinization occurring before FET.

### Ovarian stimulation and laboratory procedures

As previously described, the ovarian stimulation protocols used in 'fresh' cycles leading to oocyte retrieval and embryo cryopreservation were routine procedures at our centre [12, 13]. For ovarian stimulation, either gonadotrophin-releasing hormone agonist (GnRH-a) or GnRH antagonist (GnRH-ant) protocols were employed. Cumulus oocyte complexes was collected by transvaginal ultrasound (TVUS)-guided needle aspiration 35–36 h following hCG or GnRH-a combined hCG (dual trigger) administration. After ovum pick up, oocytes were fertilized using either conventional insemination (standard IVF) or ICSI, as indicated. Fertilization was determined 16–18 h after insemination and was judged normal when two clearly distinguishable pronuclei containing nuclei were present. Under ultrasound supervision, embryo transfer was carried out using a routine approach. The number of embryos transferred was determined on an individual basis, taking into account the patient's age, previous failed attempts, and embryo quality. Only surplus embryos or blastocysts of good quality were cryopreserved utilizing the previously described fast freezing procedure [14]. Good quality Day 3 embryos were characterized as those that reached at least the six-cell stage with < 20% fragmentation. Good quality Day 5 blastocysts were defined as having a full blastocoel cavity with trophectoderm and inner cell mass quality scores of AA, AB, BA or BB. Concerning the developmental stages of embryos and the number of embryos transferred, our center has consistently adhered to the principle of transferring one high-quality Day 3 embryos or two suboptimal Day 3 embryos or one high-quality Day 5 blastocyst.

### Frozen embryo transfer protocol

In a subsequent cycle, patients were administered hormones for endometrial preparation prior to FET. For scheduling purposes, patients with irregular ovulation or anovulation underwent suppression of their hypothalamic-pituitary-ovarian axis with oral contraceptive pills for a minimum of 21 days. There were no medical contraindications for pre-treatment with oral contraceptives. On day 3 of vaginal bleeding (after withdrawal of oral contraceptives) or spontaneous menses, patients performed a baseline TVUS and serum oestrogen, P4, luteinizing hormone, follicle stimulating hormone, and β-hCG monitoring to establish that they were in the early follicular phase of their menstrual cycle and to rule out pregnancy.

Each senior physician at our center is assigned a certain day of the week to perform the embryo transfer procedure. In most cases, this date is predetermined. Thus, the duration of endometrial preparation varies by 7 or 14 days. Nevertheless, the shortest endometrial preparation time is no less than 7 days. Patients then began oral oestrogen (France; DELPHARM Lille S.A.S.), 2 mg three times daily for 1 or 2 weeks, depending on the grouping (Group A or Group B). The purpose of oral oestrogen administration was to stimulate endometrial growth while preventing the formation of a dominant follicle. We used TVUS to assess the patients' endometrium on a regular basis, with the first ultrasound performed within 7 days of initiating oestrogen administration. Before commencing P4 supplementation, ultrasonography was performed to confirm that the endometrial thickness was more than 7 mm, and serum P4 was evaluated to rule out premature ovulation.

Once the FET's time has been determined, P4 in the form of intramuscular (Zhejiang Xianju Pharmaceutical Co., Ltd) or a combination of oral (Dydrogesterone; Abbott Biologicals B.V.) and vaginal (8% Crinone; Merck-Serono) administration was administered daily. Patient preference dictated the method of P4 supplementation. There were no medical indications to choose one regimen over the other. 3 to 5 days prior to FET, patients received intramuscular P4 or a combination of oral and vaginal P4. The vitrified-warming embryo or blastocyst was selected for transfer on the fourth or sixth day of P4 administration based on morphological grading according to the Gardner and Schoolcraft scale. Daily oestrogen and P4 medication were maintained after FET until a negative pregnancy test was reported. If a pregnancy was established, hormone treatment was maintained until the anticipated luteal-placental shift in oestrogen and P4 production, which occurred at approximately 8 to 9 weeks of gestation.

### Assessment of pregnancy outcomes

The primary outcome was the cumulative live birth rate (CLBR), which was defined as the delivery of a liveborn (> 24 weeks of gestation) using embryos obtained from the same ovarian stimulation cycle. Positive pregnancy rate, pregnancy loss rate, ectopic pregnancy rate, ongoing pregnancy rate, and live birth rate were secondary outcomes. Preeclampsia, gestational diabetes, gestational hypertension, preterm delivery, low birth weight, infants born small or large for gestational age, and congenital anomalies were among the maternal and neonatal outcomes monitored in pregnancies that lasted longer than 24 weeks. All secondary outcomes are described in Supplementary Table S1.

### Statistical analysis

Patients were categorized into two groups: Group A (7 days of oestrogen prior to P4 supplementation) and Group B (14 days of oestrogen prior to P4 supplementation). The distribution of the observations was investigated for continuous variables. The mean values and standard deviation (SD) within each group of interest were employed in the case of normal distribution (Shapiro–Wilk test). Whereas if distribution was not normal, the median and interquartile range (IQR) were reported. Categorical variables, including numerator and denominator values, are presented as either number of cases or percentages. Depending on the normality of the distribution, continuous variables were compared using an independent t-test or a Mann–Whitney U-test. The chi-squared or Fisher's exact test was used to compare categorical variables. Two-tailed alpha of 0.05 were employed in all statistical tests. All analyses were performed using SPSS 26 (SPSS Inc., Chicago, IL, USA).

To identify characteristics that may be associated with the CLBR, multivariable logistic regression analysis was performed with the CLBR as the dependent variable and duration of oestrogen administration in FET cycles (7 days versus 14 days) as the main independent variable. The potential predictors considered for the analysis were female age, body mass index (BMI), basal follicle stimulating hormone, anti-müllerian hormone, ovarian stimulation protocol, ovulation trigger, number of oocytes retrieved, embryo transfer stage, number of embryos transferred, assisted hatching, physicians of embryo transfer, and endometrial thickness prior to FET. All variables were entered into the logistic regression model simultaneously or separately. The likelihood of CLBR is presented as an odds ratio (OR) with 95% confidence interval (CI). Additionally, we performed subgroup analyses of CLBR taking into account all confounding variables, including duration of oestrogen administration in FET cycles. In our study, we also used binary multivariate logistic regression analysis to assess the association between duration of oestrogen administration and pregnancy outcomes per FET after adjusting for 7 confounding variables such as female age (< 37 yrs., ≥ 37 yrs.), BMI (< 24 kg/m^2^, ≥ 24 kg/m^2^), number of transferred embryos (single embryo transfer (SET), double embryo transfer (DET)), Day of FET (day 3, day 5), assisted hatching (yes, no), physicians of embryo transfer (A, B, C, and D) and endometrial thickness before FET. We calculated crude OR and adjusted OR with 95% CI.

## Results

### Baseline characteristics of the study population

**Fig. 1:**
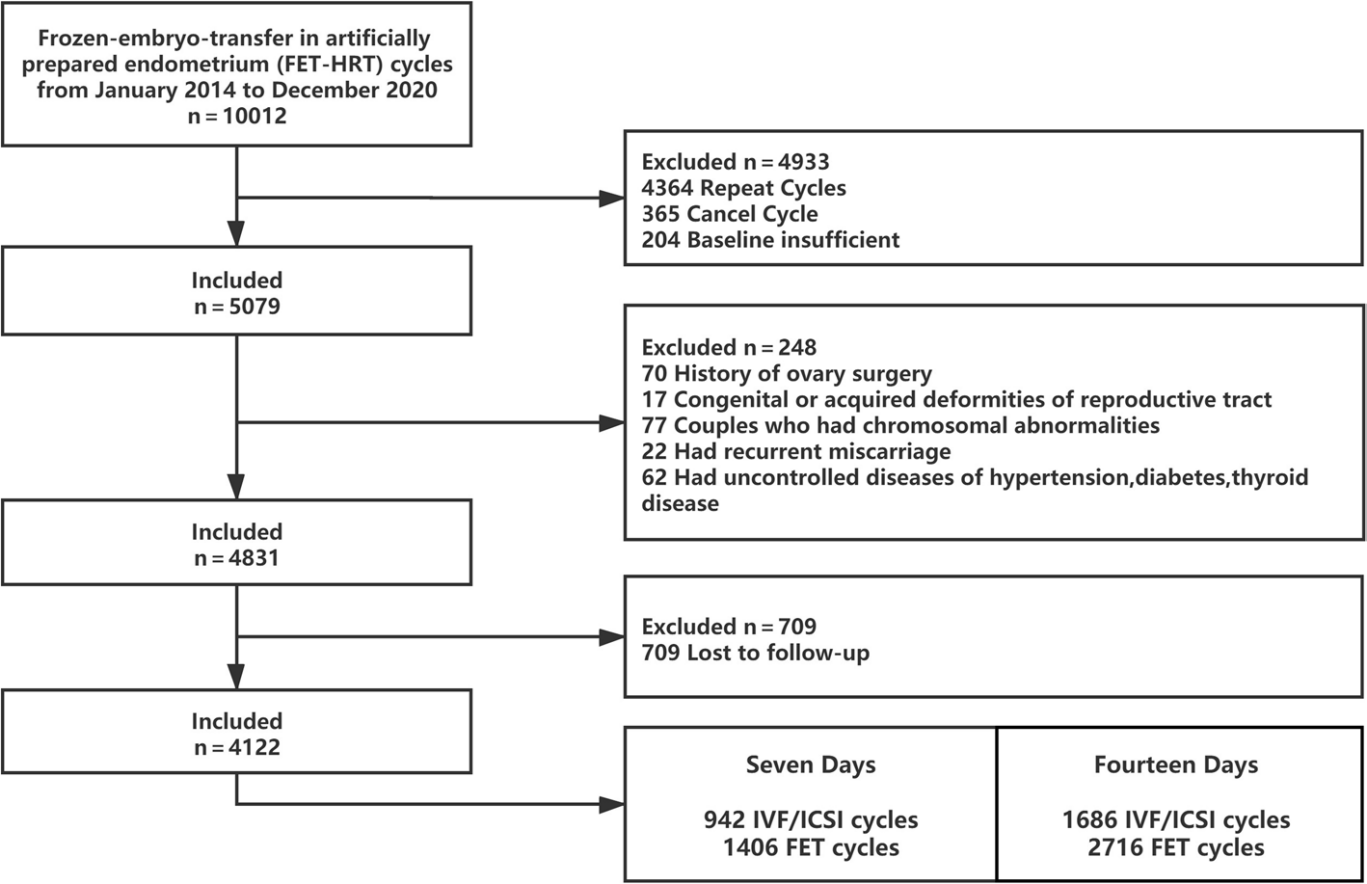
The flow chart of study enrollment

In total, 2628 patients (4142 FET cycles) were included in this study (Fig. 1) and were divided into two groups. Group A (*n* = 1406) used 7 days of oestrogen for endometrial preparation and Group B (*n* = 2716) used 14 days of oestrogen for endometrial preparation.

Baseline characteristics are presented in Table 1. Overall, there were no statistical differences between the two groups, except for a slightly longer duration of infertility in the group A than those in the group B: (3.5 (2, 4) versus 3.3 (2, 4), respectively, *P* = 0.023).

**Table 1:** Baseline characteristics, ovarian stimulation outcomes and cycle features of FETs

Characteristic	Seven Days (Group A)	Fourteen Days (Group B)	*P-value*
**Patients**	**942**	**1686**	
**FET cycles**	**1406**	**2716**	
Female age at oocyte retrieval (years; mean (SD))	31.0 (4.3)	31.2 (4.6)	*0.480*
Etiology of infertility			*0.542*
Tubal factor	509 (54.0)	923 (54.7)	
Male factor	257 (27.3)	448 (26.6)	
PCOS	126 (13.4)	206 (12.2)	
Endometriosis	50 (5.3)	109 (6.5)	
Duration of infertility (years; mean (IQR))	3.5 (2, 4)	3.3 (2, 4)	*0.023*
Nulliparous	491 (52.1)	885 (52.5)	*0.856*
Gravidity (median (IQR))	0 (0, 1)	1 (0, 1)	*0.808*
Parity (median (IQR))	0 (0, 0)	0 (0, 0)	*0.261*
BMI (kg/m^2^; mean (SD))	23.9 (3.9)	23.7 (4.0)	*0.529*
AMH (ng/ml; median (IQR))	3.3 (1.8, 5.5)	3.3 (1.8, 5.5)	*0.890*
Basic FSH (mIU/ml; mean (SD))	6.7 (2.4)	6.8 (2.3)	*0.258*
Basic LH (mIU/ml; mean (SD))	6.0 (4.2)	6.1 (4.9)	*0.950*
Basic oestradiol (pg/ml; median (IQR))	27.5 (20.8, 38.0)	26.9 (20.8, 37.9)	*0.642*
Ovarian stimulation protocol			*0.019*
GnRH-a long protocol	765 (81.2)	1429 (84.8)	
GnRH-ant protocol	177 (18.8)	257 (15.2)	
No of days of COS (mean (SD))	11.6 (2.7)	11.6 (2.7)	*0.781*
Total Gn dose administered (IU; mean (SD))	2530.5 (983)	2509.2 (975.9)	*0.647*
Serum LH on trigger day (mIU/ml; median (IQR))	1.3 (0.8, 2.2)	1.3 (0.8, 2.3)	*0.439*
Serum estradiol on trigger day (pg/ml; median (IQR))	4800 (2956, 4901)	4800 (2858, 4936)	*0.055*
Serum progesterone on trigger day (ng/ml; median (IQR))	1.2 (0.9, 1.8)	1.3 (0.9, 1.8)	*0.819*
Ovulation trigger protocol			*0.056*
hCG	875 (92.9)	1597 (94.7)	
GnRH-a and hCG (dual trigger)	67 (7.1)	89 (5.3)	
Method of fertilization			*0.693*
IVF	685 (72.7)	1238 (73.4)	
ICSI	257 (27.3)	448 (26.6)	
No of oocytes retrieved (mean (SD))	16.5 (8.0)	16.9 (8.5)	*0.413*
2PN fertilization (median (IQR))	9 (6, 13)	10 (6, 14)	*0.127*
No of embryos available for transfer (mean (IQR))	5.1 (2, 7)	5.5 (3, 8)	*0.026*
Blastocysts available for transfer	101 (10.7)	213 (12.6)	*0.147*
No of high-quality embryos (mean (IQR))	1.8 (0, 3)	2.1 (0, 3)	*0.013*
Ovarian stimulation cycle outcomes			*0.356*
Cycle with fresh embryo transfer	116 (12.3)	229 (13.6)	
Cycle with freeze-all strategy	826 (87.7)	1457 (86.4)	
Day of FET			< *0.001*
Day 3	1305 (92.8)	2287 (84.2)	
Day 5	101 (7.2)	429 (15.8)	
No of frozen thawed embryos transferred			< *0.001*
SET	288 (20.5)	807 (29.7)	
DET	1118 (79.5)	1909 (70.3)	
Assisted hatching			*0.277*
Yes	191 (13.6)	403 (14.8)	
No	1215 (86.4)	2313 (85.2)	
Endometrial thickness prior to FET (mm; mean (SD))	10.4 (1.9)	10.3 (1.9)	*0.281*
Physicians of embryo transfer			*0.002*
Physician A	657 (46.7)	1111 (40.9)	
Physician B	265 (18.9)	520 (19.1)	
Physician C	276 (19.6)	605 (22.3)	
Physician D	208 (14.8)	480 (17.7)	

*Abbreviation*: *FET* Frozen embryo transfer, *IQR* Interquartile range, *BMI*, Body mass index, *AMH* Anti-müllerian hormone, *FSH* Follicle stimulating hormone, *LH*, Luteinizing hormone, *GnRH-a* Gonadotropin releasing hormone agonist, *GnRH-ant*, Gonadotropin releasing hormone antagonist, *IVF* In-vitro fertilization, *ICS*I Intracytoplasmic sperm injection, *2PN* Double pronuclear fertilization, *Gn* Gonadotropin, *IU* International units, *SET* Single embryo transfer, *DET* Double embryo transfer

Data are presented as numbers (%) unless otherwise noted

### Ovarian stimulation and embryological characteristics

The characteristics of the ovarian stimulation cycles were comparable for the two groups (Table 1) in terms of the number of days of ovarian stimulation, total gonadotropin dose administered, serum oestradiol, progesterone, and luteinizing hormone concentrations on trigger day, method of fertilization, ovulation trigger protocol, as well as number of oocytes retrieved. A higher percentage of GnRH-ant protocol use was noted in group A patients (18.8% vs. 15.2% for group A and group B patients, respectively; *P* = 0.008). There were no significant differences between the two groups referring to embryological characteristics (Table 1), except for more available and high-quality embryos were observed in group B (5.1 (2, 7) vs. 5.5 (3, 8) for group A and group B, *P* = 0.026 and 1.8 (0, 3) vs. 2.1 (0, 3) for group A and group B, *P* = 0.013, respectively).

### Characteristics of the FET cycles

These several characteristics were not balanced and comparable between the two groups on the day of FET (day 3, day 5), number of frozen-thawed embryos transferred (SET, DET), and physicians of embryo transfer (A, B, C, and D) (*P* < 0.001, *P* < 0.001, and *P* = 0.002, respectively). Nevertheless, other characteristics of the programmed cycles, such as endometrial thickness or whether to perform assisted hatching or not, did not differ between the two study groups. (Table 1).

### Pregnancy and birth outcomes

As shown in Table 2, the primary outcome of CLBR in the group A was non-inferior to the group B (47.6% vs. 48.8%, relative risk (RR) 0.97, 95% CI 0.90–1.06, *P* = 0.537). Moreover, no significant difference was found in the ongoing pregnancy and live birth rates between the group A and the group B (34.6% vs. 31.7%, RR 1.09, 95% CI 0.99–1.19, *P* = 0.067; 34.4% vs. 31.6%, RR 1.25, 95% CI 0.99–1.57, *P* = 0.069, respectively). In the group A, 622 of 1406 FETs (44.2%) had a higher clinical pregnancy rate compared with 1113 out of 2716 (41.0%) in the group B (RR 1.08, 95% CI 1.00–1.16, *p* = 0.044). Additionally, biochemical pregnancy loss per positive hCG test was approximately 80% higher in the group B than the group A (9.4% vs. 5.3%, *P* = 0.002). The positive pregnancy rate, clinical pregnancy loss rate, and ectopic pregnancy rate did not differ significantly between the two groups. Nonetheless, binary multivariate logistic regression analysis revealed that oestrogen administration in the programmed FET cycle (7 days vs. 14 days) was not a significant independent factor of positive pregnancy rate (OR 0.99, 95% CI 0.87–1.13, *P* = 0.863), clinical pregnancy rate (OR 0.89, 95% CI 0.78–1.02, *P* = 0.088), and live birth rate (OR 0.91, 95% CI 0.79–1.05, *P* = 0.183). (Table 3) The detailed results of secondary outcomes on maternal and perinatal complications are presented in Supplementary Table S2.

**Table 2:** Pregnancy and birth outcomes

	**Seven Days (Group A)**	**Fourteen Days (Group B)**	**Relative Risk (95% CI)**	***P*-value**
**Patients**	**942**	**1686**		
**FET cycles**	**1406**	**2716**		
Positive pregnancy per embryo transfer	657 (46.7)	1228 (45.2)	0.97 (0.92 to 1.03)	*0.355*
Clinical pregnancy per embryo transfer	622 (44.2)	1113 (41.0)	1.08 (1.00 to 1.16)	*0.044*
Biochemical pregnancy loss per positive pregnancy	35 (5.3)	115 (9.4)	0.57 (0.39 to 0.82)	*0.002*
Clinical pregnancy loss per clinical pregnancy	129 (20.7)	222 (19.9)	1.04 (0.86 to 1.26)	*0.693*
Total pregnancy loss per positive pregnancy	164 (25.0)	337 (27.4)	0.91 (0.78 to 1.07)	*0.245*
Ectopic pregnancy per positive pregnancy	9 (1.4)	32 (2.6)	0.53 (0.25 to 1.10)	*0.080*
Ongoing pregnancy per embryo transfer	486 (34.6)	862 (31.7)	1.09 (0.99 to 1.19)	*0.067*
Live birth per embryo transfer	484 (34.4)	859 (31.6)	1.25 (0.99 to 1.57)	*0.069*
Cumulative live birth per patient	448 (47.6)	823 (48.8)	0.97 (0.90 to 1.06)	*0.537*

Data are presented as numbers (%)

**Table 3:** Crude and adjusted odds ratio (or) for oestrogen priming of FETs and other potential confounders for cumulative live birth rate (CLBR)

Variable	Crude OR (95% CI)	Adjusted OR (95% CI)
**Oestrogen priming in FET cycles**		
Seven days	Reference	Reference
Fourteen days	1.05 (0.90 to 1.23)	1.04 (0.89 to 1.23)
**Female age at oocyte retrieval**		
< 37 yrs	Reference	Reference
≥ 37 yrs	0.60 (0.47 to 0.76)	0.65 (0.50 to 0.83)
**Body mass index (BMI)**		
< 24 kg/m^2^	Reference	Reference
≥ 24 kg/m^2^	0.94 (0.81 to 1.10)	0.93 (0.79 to 1.09)
**FSH on menstrual cycle days 2–3**		
≤ 10 mIU/ml	Reference	Reference
> 10 mIU/ml	0.87 (0.63 to 1.21)	1.03 (0.73 to 1.45)
**Anti-müllerian hormone**		
< 1.2 mIU/ml	Reference	Reference
≥ 1.2mIU/ml	1.41 (1.11to 1.79)	1.41 (1.10 to 1.80)
**Ovarian stimulation protocol**		
GnRH-a long protocol	Reference	Reference
GnRH-ant protocol	0.97 (0.79to 1.19)	1.09 (0.86 to 1.37)
**Ovulation trigger**		
hCG	Reference	Reference
GnRH-a and hCG (dual trigger)	1.04 (0.76 to 1.44)	0.99 (0.69 to 1.42)
**No of oocytes retrieved**		
≤ 9	Reference	Reference
10–15	1.37 (1.09 to 1.72)	1.30 (1.02 to 1.65)
≥ 16	1.58 (1.28 to 1.95)	1.46 (1.17 to 1.83)
**Embryo transfer stage**		
D3	Reference	Reference
D5	1.38 (1.10 to 1.74)	1.57 (1.16 to 2.13)
**No of embryos transferred**		
SET	Reference	Reference
DET	0.92 (0.77 to 1.09)	1.12 (0.89 to 1.40)
**Assisted hatching**		
No	Reference	Reference
Yes	1.03 (0.83 to 1.29)	0.97 (0.78 to 1.22)
**Physicians of embryo transfer**		
Physician A	Reference	Reference
Physician B	1.12 (0.92 to 1.37)	1.13 (0.93 to 1.39)
Physician C	0.93 (0.75 to 1.16)	0.95 (0.77 to 1.18)
Physician D	1.01 (0.81 to 1.26)	1.02 (0.81 to 1.28)
**Endometrial thickness prior to FET**		
< 8 mm	Reference	Reference
≥ 8 mm	1.29 (1.00 to 1.65)	1.25 (0.97 to 1.61)

### Multivariable regression and subgroup analyses of CLBR

As shown in Table 4, multivariable logistic regression analysis, allowing adjustment for relevant confounders, revealed that oestrogen administration in programmed FET cycles (7 days vs. 14 days) was not significantly associated with CLBR in the adjusted models (OR 1.04, 95% CI 0.89–1.23).

**Table 4:** Relationship between endometrial preparation duration and pregnancy outcomes per FET in different models

Pregnancy outcomes	Oestrogen priming in FET cycles	Crude model ^a^		Adjusted model ^b^	
		**OR (95% CI)**	***P* value**	**OR (95% CI)**	***P* value**
**Positive pregnancy**	Fourteen days	Reference		Reference	
	Seven days	0.98 (0.86 to 1.11)	0.751	0.99 (0.87 to 1.13)	0.863
**Clinical pregnancy**	Fourteen days	Reference		Reference	
	Seven days	0.88 (0.77 to 0.99)	0.045	0.89 (0.78 to 1.02)	0.088
**Live birth**	Fourteen days	Reference		Reference	
	Seven days	0.88 (0.77 to 1.01)	0.069	0.91 (0.79 to 1.05)	0.183

*BMI* Body mass index, *OR* Odds ratio, *CI* Confidence interval, *FET* Frozen-thawed embryo transfer

^a^No adjustments for other covariates

^b^Adjusted for female age (< 37 yrs., ≥ 37 yrs.), BMI (< 24 kg/m^2^, ≥ 24 kg/m^2^), number of transferred embryos (SET, DET), Day of FET (day 3, day 5), assisted hatching (yes, no), physicians of embryo transfer (A, B, C, and D) and endometrial thickness before FET

**Fig. 2:**
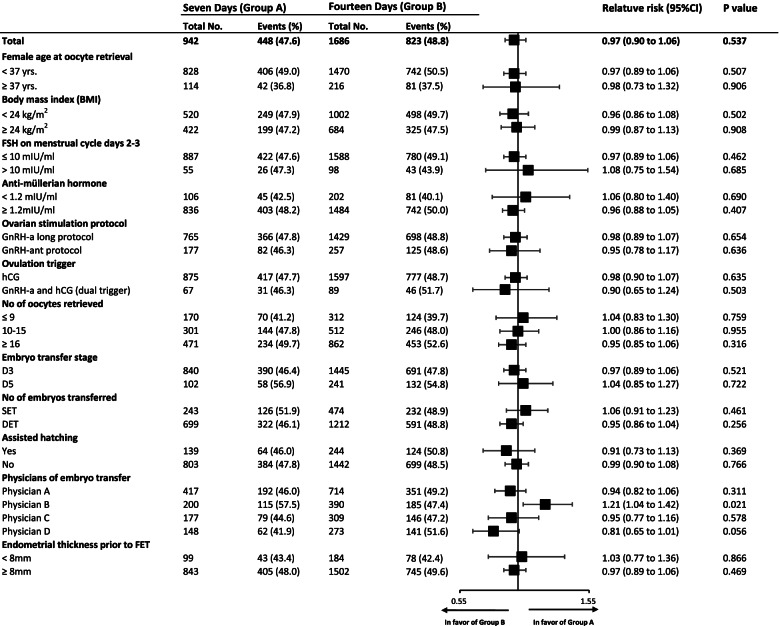
Subgroup analysis of CLBR in patients treated with oestrogen for 7 days and 14 days for endometrial preparation in programmed FET cycles

We also performed subgroup analyses of CLBR. (Fig. 2) The CLBR was significantly higher in the group A compared with those in the group B when FET procedure operations were performed by physician B (57.5% for group A vs. 47.4% for group B, *P* = 0.021), a result that deserves cautious interpretation due to the heterogeneity of this subgroup.

## Discussion

With this large retrospective study, we analyzed the effect of 7 and 14 days of oestrogen administration in programmed cycles on pregnancy outcomes in FET. The results of the study found that the clinical pregnancy rate and live birth rate as well as the cumulative live birth rate in infertile patients who underwent FET by programmed cycle preparation of the endometrium were not affected by the duration of oestrogen administration. The number of days of oestrogen use did not alter the reproductive outcome of FET, which shows that endometrial receptivity is not affected by shorter oestrogen exposure time. We therefore conclude that physicians can be flexible in scheduling FET procedures when endometrial thickness is appropriate, without being limited by the number of days of oestrogen administration.

Numerous studies have shown that the expression of endometrial genes and implantation factors are affected during implantation at higher oestrogen concentrations [15], and genes highly expressed during the implantation window of the natural cycle are down-regulated [16–18], such as endometrial integrin β3 subunit and leukaemia inhibitory factor (LIF) [16, 19]. When the appropriate concentration of oestrogen acts on the endometrium, it can make the endometrium more synchronized with the embryo [15]. However, compared with natural cycles, programmed cycles have similar reproductive outcomes although serum oestrogen levels are higher [5, 20]. In recent years, with the increasing number of FET cycles, it is particularly urgent to study the correlation between the duration of oestrogen administration in the proliferative phase and endometrial content.

In the study of donor oocyte transfer cycles, some scholars believe that a shorter duration of oestrogen can negatively affect the cycle outcome, leading to an increase in the rate of early pregnancy loss [10, 21]. Younis et al. [22] found that the duration of oestrogen administration should be controlled at 12–19 days for optimal pregnancy rates. Borini et al. [10] also found that donor oocyte transfer cycles resulted in the best reproductive outcome when the timing of oestrogen administration was controlled in the range of 11–40 days. However, some scholars believe that long-term use of oestrogen will lead to adverse pregnancy outcomes in donor oocyte transfer cycles. Michalas et al. [23] found in their study that the optimal duration of oestrogen administration was 6–11 days before progesterone addition, after which the clinical pregnancy rate decreased significantly with prolonged oestrogen exposure. Alternatively, Navot et al. [21] found a significant increase in the incidence of breakthrough bleeding in patients when oestrogen was administered for more than 40 days.

At present, few studies have addressed the effect of the number of days of continuous oestrogen administration on receiving autologous oocyte-derived frozen embryo transfer. Ying et al. [24] found that biochemical and clinical pregnancy rates were significantly higher in the later oestrogen initiation group compared with the early oestrogen initiation group, however, ongoing pregnancy rates were not significantly different between groups. However, this study did not observe the effect of duration of oestrogen on live birth rate. Sekho et al. concluded that the duration of oestrogen administration before FET was not associated with embryo implantation rate, clinical pregnancy rate, early pregnancy loss rate, and live birth rate [11]. In contrast, Bourdon et al. found in a large retrospective analysis involving 1377 autologous IVF transfers with frozen blastocysts that continuous oestrogen administration for more than 28 days before FET significantly reduced live birth rates, and that fetal birth weight and Z-score decreased with prolonged oestrogen exposure when oestrogen was administered for more than 36 days [10].

The strength of this study is the large sample size included. To date, our study provides the largest duration of oestrogen administration in programmed cycles and clinical outcomes of FET. The limitation of our study is that it is a retrospective study, conducted in a single centre, IVF/ICSI administration protocol without including PGT techniques only involves GnRH-a long protocol and GnRH-ant protocol, and FET procedure is performed by different senior doctors. Our study does not exclude the possible effects of the oestrogen administration route, although the available studies suggest that the oestrogen administration route does not affect the clinical outcome of patients [7–9]. In addition, progesterone is not administered in the same way in patients. Now, the route of clinical progesterone administration is intramuscular injection, oral and vaginal administration, or a combination regimen. Current studies suggest that the mode of progesterone administration does not affect pregnancy rates [25–27]. Furthermore, since this research is retrospective, the difference in baseline data between the two groups cannot be overlooked. Utilizing multivariate logistic regression analysis model to screen for various confounders that may impact clinical outcomes effectively avoided the statistical risk that these differences may impart. Despite these limitations, our study still provides valuable data with reference for clinicians to use flexible programmed cycle protocols.

## Conclusion

In conclusion, our study showed that oestrogen administration for 7 days and for 14 days did not affect the reproductive outcome of FET. There was no association between duration of oestrogen use and live birth rate. This provides a more flexible option for the administration regimen of FET oestrogen under programmed cycles, and, without affecting clinical outcomes, reduces patient time and economic costs to some extent. However, because this study still has some limitations and contradicts some previous findings, more prospective clinical trials need to be designed to further evaluate the effect of the number of days of oestrogen administration in programmed cycles on the clinical outcomes of FET. In addition, previous studies have found that there is a correlation between oestrogen exposure time and delivery, premature delivery rate and fetal weight [10], but there are few relevant studies, and more detailed prospective studies involving obstetric follow-up content need to be designed to further explore their relationship and provide a reference for clinical practice.

## Supplementary Information

**Additional file 1.**
